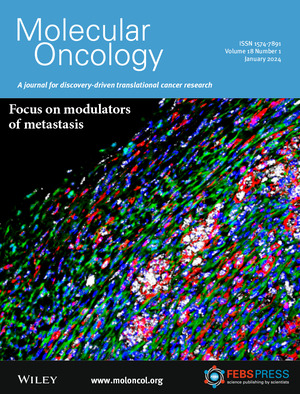# Issue Information

**DOI:** 10.1002/1878-0261.13455

**Published:** 2024-01-04

## Abstract

The issue content is focused on the various biological processes guiding cancer spread and modulators of metastasis. A microscopy image showing incorporation of cancer cell‐derived exosomes into macrophages of milky spots, isolated from mesentery sheets is displayed at the cover. Read the full article by Misato Horie *et al*. in pp. 21–43.